# Pediatric case of trichilemmal cyst arising on the face^⋆^

**DOI:** 10.1016/j.abd.2022.05.012

**Published:** 2023-09-16

**Authors:** Mai Endo, Toshiyuki Yamamoto

**Affiliations:** Department of Dermatology, Fukushima Medical University, Fukushima, Japan

Dear Editor,

Trichilemmal cyst is sometimes seen in the scalp of adults. We herein describe a rare case of a trichilemmal cyst arising on the forehead of a child.

A 9-year-old boy visited our hospital, complaining of a nodule above the left eyebrow that had increased in size over the previous year. He had no past medical history, and he and his parents denied any prior triggering events such as trauma on this site. Physical examination showed a 7 × 5 mm, normal skin-colored, slightly dome-shaped subcutaneous nodule (Fig. 1A). Laboratory examination was normal. The nodule was surgically removed under local anesthesia. Histopathological examination revealed a relatively well-circumscribed cystic structure located in the subcutaneous tissue (Fig. 1B). The cyst was filled with acidophilic amorphous substances, and the cyst walls consisted of epithelial cells without forming granular cell layers (Fig. 1C). The serial sections of histopathology showed cholesterin crystals and foreign body giant cells within and around the cyst (Fig. 1D). After the surgery was performed, 10 years have passed without local recurrence.

**Figure 1:**
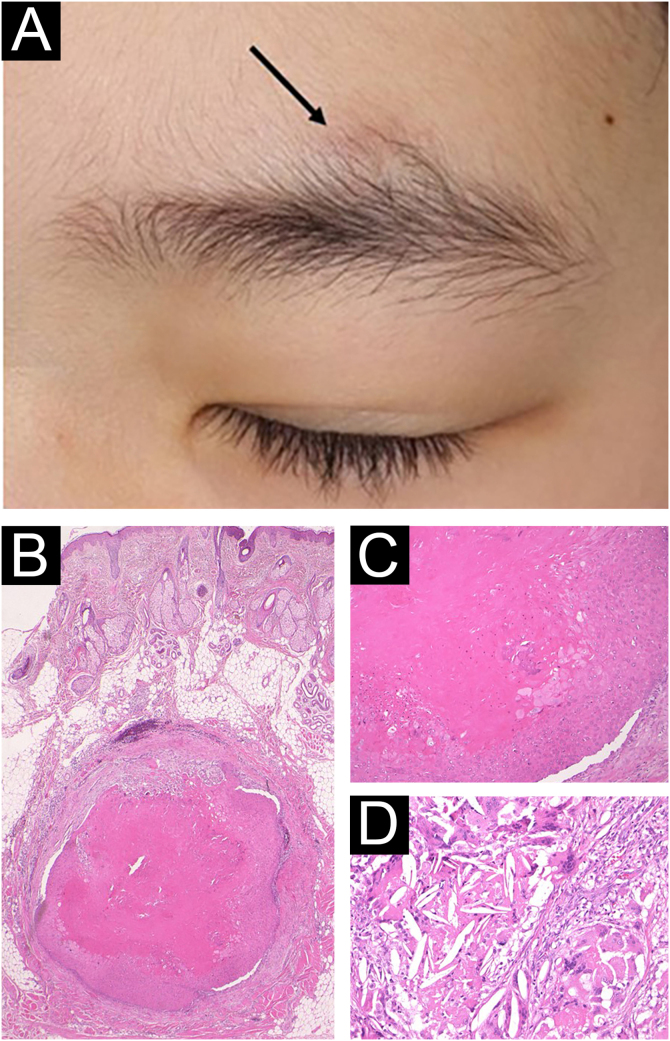
(A) A slightly dome-shaped subcutaneous nodule above the left eyebrow (arrow). (B) Histopathological examination showing a cystic structure located in the subcutaneous tissue (Hematoxylin & eosin, ×20). (C) Higher magnification shows that the cyst walls keratinize towards the lumen without forming granular cell layers (Hematoxylin & eosin, ×200). (D) Cholesterin crystals and foreign body giant cells (Hematoxylin & eosin, ×200).

We diagnosed the case as a trichilemmal cyst based on the histopathological features of well-defined cystic structures consisting of epithelial cells showing trichilemmal keratinization. Cholesterin crystals and cholesterin clefts, which are often observed in epidermal cysts, were observed in the present case, but those histopathological features are not diagnostic. The presence of foreign body granuloma may suggest previous partial ruptures of trichilemmal cysts. The cyst wall was not adjacent to sebaceous glands, and hair follicles and hair shafts were not observed in and around the cysts. Moreover, the subcutaneous nodule did not exist at birth, and thus dermoid cyst was excluded. The trichilemmal cyst is a benign adnexal tumor that arises from the outer root sheath of a hair follicle. It usually presents as an asymptomatic firm nodule, which at times can be slightly painful. It is mainly seen in areas bearing hair follicles, mostly on the scalp. Middle-aged females are more commonly affected.^1^ The onset of a trichilemmal cyst in a young boy is rare. To our knowledge, only 3 cases have been reported that developed trichilemmal cysts under the age of 10, including the present case.^2^, ^3^ Clinical findings of these cases are shown in Table 1.^2^, ^3^ The thigh, penis, and eyebrow were involved, which were rare sites. Our patient is now the youngest among the reported Japanese cases. On the other hand, the youngest case was in a 5-year-old male, who developed a trichilemmal cyst on the penis after hypospadias repair.^3^ The authors speculated that the distal hypospadias repair had triggered squamous metaplasia with keratinization, leading to the development of a trichilemmal cyst in a non-hair-bearing area of the body.

**Table 1:** Summary of the reported cases of pediatric trichilemmal cyst

Authors	Age/sex	Site	Size	Clinical features	Color
Imamura H, et al.^2^	10/male	Flexor aspect of thigh	About 15 × 20 mm	Elastic, soft, non-tender nodule	Slightly blue
Madan S, Joshi R.^3^	5/male	Ventral aspect of the frenulum of the penis	15 × 16 mm	Soft, cystic, smooth-surfaced, elastic, non-tender, and relatively mobile mass	Unidentified
Our case	9/male	Above eyebrow	7 × 5 mm	Slightly dome-shaped, non-tender subcutaneous nodule	Slightly red

In our department, 25 cases including the present case were diagnosed as trichilemmal cysts over the past 10 years, with only 1 pediatric case (the present case). The patients consisted of 12 males and 13 females, and the mean age was 49 years. The involved sites were most frequently observed in the scalp (n = 16), followed by the face (7), abdomen (1), and forearm (1). Among the facial lesions, 2 were observed in the eyebrow, 2 were observed in the upper and lower eyelids, 2 were observed on the forehead, 1 was observed in the cheek. Trichilemmal cyst is one of the nodules arising on the head and neck, which rarely involves children.

## Financial support

None declared.

## Authors' contributions

Mai Endo: Design of the study; Writing of the manuscript; data collection, analysis and interpretation; review and approval of the final version of the manuscript.

Toshiyuki Yamamoto: Design of the study; writing of the manuscript; data collection, analysis, and interpretation; review and approval of the final version of the manuscript.

## Conflicts of interest

None declared.

## References

[1] Jha A.K., Sinha R., Prasad S., Kumar S. (2015). Multiple trichilemmal cysts of the scalp in a young male. Int J Trichol..

[2] Imamura H., Izumi T., Kimura S. (1997). Two cases of trichilemmal cyst on the thigh. Jpn J Clin Dermatol (in Japanese)..

[3] Madan S., Joshi R. (2015). Trichilemmal cyst of the penis in a paediatric patient. Sultan Qaboos Univ Med J..

